# A secure remote user authentication scheme for 6LoWPAN-based Internet of Things

**DOI:** 10.1371/journal.pone.0258279

**Published:** 2021-11-08

**Authors:** Ghulam Abbas, Muhammad Tanveer, Ziaul Haq Abbas, Muhammad Waqas, Thar Baker, Dhiya Al-Jumeily OBE

**Affiliations:** 1 Faculty of Computer Science and Engineering, GIK Institute of Engineering Sciences and Technology, Topi, Pakistan; 2 Telecommunications and Networking Research Center, GIK Institute of Engineering Sciences and Technology, Topi, Pakistan; 3 Faculty of Electrical Engineering, GIK Institute of Engineering Sciences and Technology, Topi, Pakistan; 4 Engineering Research Center of Intelligent Perception and Autonomous Control, Faculty of Information Technology, Beijing University of Technology, Beijing, China; 5 Department of Computer Science, University of Sharjah, Sharjah, United Arab Emirates; 6 School of Computer Science and Mathematics, Liverpool John Moores University, Liverpool, United Kingdom; University College of Engineering Tindivanam, INDIA

## Abstract

One of the significant challenges in the Internet of Things (IoT) is the provisioning of guaranteed security and privacy, considering the fact that IoT devices are resource-limited. Oftentimes, in IoT applications, remote users need to obtain real-time data, with guaranteed security and privacy, from resource-limited network nodes through the public Internet. For this purpose, the users need to establish a secure link with the network nodes. Though the IPv6 over low-power wireless personal area networks (6LoWPAN) adaptation layer standard offers IPv6 compatibility for resource-limited wireless networks, the fundamental 6LoWPAN structure ignores security and privacy characteristics. Thus, there is a pressing need to design a resource-efficient authenticated key exchange (AKE) scheme for ensuring secure communication in 6LoWPAN-based resource-limited networks. This paper proposes a resource-efficient secure remote user authentication scheme for 6LoWPAN-based IoT networks, called SRUA-IoT. SRUA-IoT achieves the authentication of remote users and enables the users and network entities to establish private session keys between themselves for indecipherable communication. To this end, SRUA-IoT uses a secure hash algorithm, exclusive-OR operation, and symmetric encryption primitive. We prove through informal security analysis that SRUA-IoT is secured against a variety of malicious attacks. We also prove the security strength of SRUA-IoT through formal security analysis conducted by employing the random oracle model. Additionally, we prove through Scyther-based validation that SRUA-IoT is resilient against various attacks. Likewise, we demonstrate that SRUA-IoT reduces the computational cost of the nodes and communication overheads of the network.

## 1 Introduction

Low-power wireless personal area networks (LoWPANs) have found numerous applications in the Internet of Things (IoT) [1]. LoWPAN devices are amenable with IEEE 802.15.4 and are constricted in power, communication, data rate, and storage resources [2]. IEEE 802.15.4-enabled LoWPAN devices are deployed in various real-world applications, such as home automation, healthcare systems, security surveillance, smart grids, and industrial motoring. To provide Internet connectivity to a large number of devices deployed in a particular IoT environment, the IPv6 protocol is considered the most accordant solution because of its larger address space to render a unique IP address to each sensor node. By using IPv6 addressing, sensor nodes can transmit sensed information to other devices or to a central location through the public Internet.

To support large-scale connectivity for IoT, the Internet Engineering Task Force has designed IPv6-over-LoWPAN (6LoWPAN) adaptation layer to render packet fragmentation, reassembly, and encapsulation features for IEEE 802.15.4-based LoWPAN networks [3, 4]. Since LoWPAN devices collect information and send to a designated location via the public Internet, it is imperative for LoWPAN applications to provide security and privacy. However, the basic 6LoWPAN design does not provide security and privacy features to preclude an unauthorized network entity from procuring the collected information and to prevent illegitimate users from accessing the 6LoWPAN network resources [5–9].

6LoWPANs encounter the same security attacks as the traditional networks. These include denial-of-service (DoS), replay, user/server impersonation (UI/SI), man-in-the-middle (MITM), identity guessing (IG), user anonymity (UA), user/device impersonation (UI/DI), stolen smart card/device (SSC/SSD), and ephemeral secret leakage (ESL) attacks. However, due to the resource-constricted nature of 6LoWPANs and the inadequacy of organized network architectures, securing 6LoWPAN becomes more challenging [10]. Authentication, availability, integrity, data freshness, and confidentiality are imperative security provisions in 6LoWPANs. Confidentiality guarantees secure data transmission between authorized users and servers. Authentication and key establishment (AKE) is the mechanism to identify devices’ and users’ legitimacy in 6LoWPANs [11] and to set up a secret session key (SK) for encrypted communication. Therefore, a lightweight AKE mechanism becomes imperative for securing the network [12–20].

### 1.1 Related work

An overview of the existing AKE schemes for 6LoWPAN-based IoT networks and their limitations is presented in S1 Table, which shows that no existing scheme can withstand all known attacks. Pandi *et al*. [42] propounded an authentication scheme for vehicular ad-hoc networks (VANETs) to enable the network entities to communicate securely. The scheme presented by Pandi *et al*. is efficient in terms of certificate computation while preserving privacy of the entities. Pandi *et al*. [43] presented an AKE scheme for IoT-based wireless body area networks (WBANs), which is computationally less expensive and ensures secure communication. Azees *et al*. [44] propounded an anonymous authentication scheme for WBANs, which is capable of resisting various covert security attacks while requiring fewer resources. Azees *et al*. [45] presented a blockchain based authentication scheme for VANETs, which is capable of resisting different security attacks and renders secure communication in VANETs.

The authors in [34] propounded a multi-factor AKE scheme for the IoT environment. The AKE scheme proffered in [34] uses a lightweight hash function along with advanced encryption standard (AES). However, the scheme is unable to restrain SSD, DoS, replay, and sensor node (SN) capture attacks. A Chinese remainder theorem-based authentication scheme is presented in [23], which cannot resist replay attack and does not provide strong privacy. In addition, a signature and certificate-based computationally efficient authentication scheme for VANETs is presented in [46]. The authors in [47] propounded a resource-efficient AKE scheme for the IoT environment by utilizing hash function and XOR operation. However, the AKE scheme presented in [47] is prone to SSD, stolen verifier, UI, and UA attacks and is unable to ensure SN’s anonymity. An AKE scheme is propounded in [48] for mobile networks. The scheme proposed in [48] is resource-efficient and is suitable for mobile networks. A cosine similarity-based AKE scheme for the IoT environment is proposed in [49]. Furthermore, to enable security and privacy in different IoT-based networks, various AKE schemes are reported in the exiting literature [19, 50–66].

Additionally, the security analysis of an eminent AKE scheme presented in [31] is given at S1 Appendix. We have thoroughly analyzed the scheme and demonstrate that it is unsafe against de-synchronization attack and does not provide a revocation phase (RP). In [31], gateway broadcasts the authentication message to all sensor nodes deployed in the network, and a user does not specify the sensor node from which it is going to procure the information. Thus, all the sensor nodes in the network process the received message, which causes an extra computational overhead for every node.

### 1.2 Research contributions

This paper presents a resource-efficient secure remote user authentication scheme for 6LoWPAN-based IoT networks (SRUA-IoT). The proposed scheme performs user authorization before procuring real-time data from sensors stationed in the 6LoWPAN-based IoT networks. The scheme employs a lightweight secure hash algorithm (SHA-160) and advanced encryption standard (AES-192) to accomplish the AKE process and makes the following contributions.

1. SRUA-IoT is an AES and hash function based remote user AKE scheme for 6LoWPAN-based IoT networks, which renders user revocation and password change phases. Besides, SRUA-IoT ensures the legitimacy of remote users (RUs) to access real-time information from a sensor node while ensuring the privacy and anonymity of RUs. An RU indicates to the gateway a particular sensor node for procuring real-time information, which reduces the unnecessary computational overhead.
2. SK’s security in SRUA-IoT is corroborated using random oracle model (ROM). Informal security validation illustrates that SRUA-IoT is protected against SSC, de-synchronization, replay, and DoS attacks. In addition, Scyther tool analysis illustrates that the proposed scheme is protected.
3. We demonstrate that SRUA-IoT renders enhanced security functionalities aside from its low storage, computational, and communication costs, as compared to well-known AKE schemes.

### 1.3 Paper organization

The remainder of this paper is organized as follows. The system model is presented in Section 2. The proposed SRUA-IoT scheme is elaborated in Section 3. Security analysis is presented in Section 4. Performance evaluation of SRUA-IoT is detailed in Section 5. Finally, the paper is concluded in Section 6.

## 2 System model

The network model consists of a gateway *GW*, a registration center (RC), and remoter users (*RU*_*y*_|*y* = 1, 2, 3, ⋯, *N*). In the SH environment, sensor nodes (*SN*_*x*_|*x* = 1, 2, 3, ⋯, *n*) are deployed to monitor various processes. *SN*_*x*_ collect critical information and forward to the server stationed at RC. RC is responsible for the deployment of *SN*_*x*_ and implementing various access control policies in SH. Before procuring real-time information from *SN*_*x*_, it is necessary for *RU*_*y*_ to register with RC. After registration, *RU*_*y*_ can access the network resources and the allocated *SN*_*x*_. It is assumed that all network nodes are time synchronized.

The well-established Dolev Yao (DY) threat model [67] is employed, wherein an adversary A can intercept communications between two network entities communicating via a public channel. A can modify the intercepted messages or use the message for various malicious purposes. A can procure the secret credentials stored in a sensor node’s memory. Furthermore, A can obtain *RU*_*y*_’s smart device *SD*_*y*_ and can extract secret credentials form *SD*_*y*_ to execute various security attacks.

*RU*_*y*_ needs to communicate with *SN*_*x*_ to securely procure the real-time information collected by *SN*_*x*_. Therefore, an AKE scheme is imperative for secure and reliable communications between *RU*_*y*_ and *SN*_*x*_. To achieve reliable and secure communication, the following section presents an *RU*_*y*_ AKE scheme, called SRUA-IoT.

## 3 The proposed SRUA-IoT scheme

SRUA-IoT seeks to ensure reliable and secure access to 6LoWPAN network resources. The scheme first verifies the authenticity of *RU*_*y*_ and then establishes a secret SK for encrypted communication by employing a lightweight hash function and AES-192 during the AKE process. SHA is an irreversible function, which means that it is impossible to derive the input from the output of SHA-160. Moreover, SHA-160 is a collision resistance function, which means that the output of SHA-160 can never be the same for different inputs. AES-192 is used as the encryption and decryption scheme in SRUA-IoT. SRUA-IoT is composed of seven phases, which are presented in the following subsections. S2 Table lists the notations used in this paper.

### 3.1 Sensor node deployment phase

RC assigns various secret credentials to *SN*_*x*_ before its deployment in the 6LoWPAN network. Moreover, RC selects a *GW*’s secret Key (*GK*) of 512 bits and a unique identity *ID*_*G*_. Both *GK* and *ID*_*G*_ are known only to *GW*. RC executes the following steps to accomplish the sensor node deployment (SND) phase.

#### 3.1.1 Step SND-1

RC picks a unique $ID_{SN_{x}}$ and $PID_{SN_{x}}$ each of size 80 bits. Moreover, RC selects a random number *R*_*x*_ and computes a temporary secret (TS) for *SN*_*x*_ as *A*_*e*_ = *H*(*GK* ∥ *R*_*x*_∥ *ID*_*G*_), and $TS_{SN_{x}}=A_{e}^{a}⊕A_{e}^{b}$, where $A_{e}^{a}$ and $A_{e}^{b}$ are two chunks of *A*_*e*_, each of size 80 bits.

#### 3.1.2 Step SND-2

RC stores the credentials {$ID_{SN_{x}}$, $PID_{SN_{x}}$, $TS_{SN_{x}}$} in the memory of *SN*_*x*_ before its deployment.

### 3.2 Remote user registration phase

It is imperative for RC to register *RU*_*y*_ before providing access to the 6LoWPAN network resources. RC assigns different secret credentials and a list of *SN*_*x*_ to *RU*_*y*_. RC executes the following steps to perform the remote user registration (RUR) phase.

#### 3.2.1 Step RUR-1

*RU*_*y*_ selects a distinct identity $ID_{RU_{y}}$ and computes $HID_{y}=H(ID_{RU_{y}})$. Moreover, *RU*_*y*_ contrives a registration message $ME_{1}^{r}:\left\{HID_{y}\right\}$ and dispatches $ME_{1}^{r}$ to RC via a protected channel.

#### 3.2.2 Step RUR-2

RC selects a distinct pseudonym *PID*^*x*^ for *RU*_*y*_ and calculates *A*_*q*_ = *H*(*GK* ∥ *ID*_*G*_), and *A*_*x*_ = *H*(*HID* ∥ *ID*_*G*_ ∥ *GK*). RC determines a TS credential for *RU*_*y*_ by dividing *A*_*x*_ into two equal parts, namely, $A_{x}^{a}$ and $A_{x}^{b}$, each of size 80 bits, and computes $TS_{RU_{y}}=A_{x}^{a}⊕A_{x}^{b}$. Moreover, RC computes the revocation parameter (ReP) as *B*_*x*_ = *A*_*q*_ ⊕ *HID*_*y*_ and $RP_{RU_{y}}=B_{x}^{a}⊕B_{x}^{b}$, where $B_{x}^{a}$ and $B_{x}^{b}$ are two chunks of *B*_*x*_. Besides, RC assigns a list of *SN*_*x*_ to be accessed by *RU*_*y*_. Furthermore, RC computes encryption key as *EK* = (*A*_*q*_ ∥ [*A*_*q*_]^32^), where [*A*_*q*_]^32^ are the first 32 bits of *A*_*q*_ (to make the size of *EK* 192 bits). In addition, RC derives $CT_{RU_{y}}=E_{EK}\{TS_{RU_{y}},PID_{SN_{x}},TS_{SN_{x}}\}$ by using AES-192, and stores a list of credentials {*PID*^*x*^, $RP_{RU_{y}}$, $CT_{RU_{y}}$} in *GW*’s memory. Finally, RC fabricates a message $ME_{2}^{r}:\{PID^{x},TS_{RU_{y}},PID_{SN_{x}}\}$ and sends $ME_{2}^{r}$ to *RU*_*y*_ securely.

#### 3.2.3 Step RUR-3

After procuring $ME_{2}^{r}$ from RC, *RU*_*y*_ supplies its $ID_{RU_{y}}$, password $PS_{RU_{y}}$ and $B_{RU_{y}}$ at the interface of smart device *SD*_*y*_ and computes $\left(\beta_{k},Rp)=Gen(B_{RU_{y}}\right)$ by using fuzzy extractor (*FE*). *FE* consist of two functions. The first one is *Gen*(.), which is a probabilistic function that takes bio-metric information $B_{RU_{y}}$ of *RU*_*y*_ and produces two output parameters, namely, secret bio-metric key *β*_*k*_ and reproduction parameter *Rp*. The second function of *FE* is *Rep*(.), which is a deterministic function that takes *Rp* and $B_{RU_{y}}$ to reproduce *β*_*k*_. Moreover, *SD*_*y*_ calculates $Z_{x}=H(PID^{x}∥TS_{RU_{y}}∥PID_{SN_{x}})$, $Z_{y}=H(ID_{RU_{y}}∥PS_{RU_{y}}∥\beta_{k})$, and encryption key *EK*_*y*_ = (*Z*_*y*_ ∥ [*Z*_*y*_]^32^), where [*Z*_*y*_]^32^ are the first 32 bits of *Z*_*y*_ to create *EK*_*y*_ of size 192 bits. Furthermore, *SD*_*y*_ calculates $CT_{lo}=E_{EK_{y}}\{PID^{x},TS_{RU_{y}},CT_{RU_{y}}\}$ by using AES-192. In addition, *SD*_*y*_ computes authentication parameter as $Auth_{y}=H(ID_{RU_{y}}∥PS_{RU_{y}}∥\beta_{k}∥Z_{x})$.

#### 3.2.4 Step RUR-4

Finally, *SD*_*y*_ stores the list of credentials {*CT*_*lo*_, *Auth*_*y*_, *Rp*, *Gen*(.), *Rep*(.), *Et*} in its memory and deletes all other parameters.

### 3.3 RU AKE phase

To access and communicate with the deployed 6LoWPAN based devices, it is necessary for *RU*_*y*_ to register itself with RC. RC allocates a list of secret credentials and devices to *RU*_*y*_ at the time of registration. After authorizing *RU*_*y*_’s legitimacy, RC allows *RU*_*y*_ to access the specified devices deployed in the network. After getting authenticated by RC, *RU*_*y*_ and *SN*_*x*_ set up an SK for reliable and secure communication. The following steps elaborate RU AKE phase (RAP).

#### 3.3.1 Step RAP-1

*SD*_*y*_ receives the secret credentials $PS_{RU_{y}}$, $ID_{RU_{y}}$, and $B_{RU_{y}}$, and computes $\beta_{k}=Rep(B_{RU_{y}},Rp)$ and $Z_{y}=H(ID_{RU_{y}}∥PS_{RU_{y}}∥\beta_{k})$. In addition, *SD*_*y*_ computes the decryption key *DK*_*lo*_ as *DK*_*lo*_ = (*Z*_*y*_ ∥ [*Z*_*y*_]^32^), where [*Z*_*y*_]^32^ are the first 32 bits of *Z*_*y*_ to make *DK*_*lo*_ of size 192 bits. Moreover, *SD*_*y*_ computes $PT_{lo}=D_{DK_{lo}}\{CT_{lo}\}$, where *CT*_*lo*_ is the ciphertext stored in *SD*_*y*_, and retrieves $PT_{lo}=\{PID^{x},TS_{RU_{y}},PID_{SN_{x}}\}$. Furthermore, *SD*_*y*_ calculates $Z_{x}^{lo}=H(PID^{x}∥TS_{RU_{y}}∥PID_{SN_{x}})$, and authentication parameter $Auth_{lo}=H(ID_{RU_{y}}∥PS_{RU_{y}}∥\beta_{k}∥Z_{x})$. Finally, *SD*_*y*_ checks *Auth*_*y*_ = *Auth*_*lo*_ to perform local authentication. If the condition holds, *SD*_*y*_ continues the AKE process.

#### 3.3.2 Step RAP-2

After performing the local authentication, *SD*_*y*_ chooses *T*_*x*_ of size 32 bits, and *R*_1_ of size 80 bits. *SD*_*y*_ calculates $G_{1}=\left(R_{1}∥PID_{SN_{x}}\right)⊕H\left(TS_{RU_{y}}∥T_{x}\right)$ and $Auth_{a1}=H\left(PID_{}^{x}∥PID_{SN_{x}}∥R_{1}∥TS_{RU_{y}}\right)$. Furthermore, *SD*_*y*_ contrives a message *ME*_*a*_: {*T*_*x*_, *PID*^*x*^, *G*_1_, *Auth*_*a*1_} and dispatches it to *GW* via an open communication channel.

#### 3.3.3 Step RAP-3

Upon procuring *ME*_*a*_ from *SD*_*y*_, *GW* verifies the validity of timestamp by validating the condition *TD*_*x*_ ≥ |*Tr* − *T*_*x*_|, where *TD*_*x*_ is maximum tolerable packet time delay, *Tr* is the receiving time of *ME*_*a*_, and *T*_*x*_ is fabrication time of *ME*_*a*_. If *ME*_*a*_ receives at the *GW* within the maximum allowed time delay limit, *GW* considers *ME*_*a*_ to be a licit and fresh message and continues the AKE phase. *GW* picks *PID*^*x*^ from the received *ME*_*a*_ and looks up *PID*^*x*^ in *GW*’s memory. If found, *GW* extracts the list of credentials {*PID*^*x*^, $RP_{RU_{y}}$, $CT_{RU_{y}}$} related to *PID*^*x*^. In addition, *GW* calculates *DK* as *M*_1_ = *H*(*GK* ∥ *ID*_*G*_) and *DK* = (*M*_1_ ∥ [*M*_1_]^32^). Moreover, *GW* computes $PT_{1}=D_{DK}\left\{CT_{RU_{y}}\right\}$ by using AES-192, and procures secret credentials {$TS_{RU_{y}}$, ($PID_{SN_{x}}$, $TS_{SN_{x}}$)} from *PT*_1_. Furthermore, *GW* obtains *R*_1_ and $PID_{SN_{x}}$ by computing $\left(R_{1}∥PID_{SN_{x}})=G_{1}⊕H(TS_{RU_{y}}∥T_{x}\right)$. To validate the authenticity of *ME*_*a*_, *GW* calculates $Auth_{a2}=H(PID^{x}∥PID_{SN_{x}}∥R_{1}∥TS_{RU_{y}})$ and verifies the condition *Auth*_*a*1_ = *Auth*_*a*2_. If the condition holds, *GW* continues the execution of the AKE process.

#### 3.3.4 Step RAP-4

After validating the authenticity of *ME*_*a*_, *GW* picks a timestamp *T*_*y*_ and random number *R*_2_, and computes $W_{1}=H(R_{1}∥TS_{RU_{y}}∥PID^{x})$, where *W*_1_ is obtained using hash of the parameters, including *R*_1_, $TS_{RU_{y}}$, and *PID*^*x*^. *GW* calculates the update parameter (UP) as $UP=W_{1}^{a}⊕W_{1}^{b}$, where $W_{1}^{a}$ and $W_{1}^{b}$ are obtained by dividing *W*_1_ into two equal chunks of 80 bits each. Besides, *GW* computes *PID*^*x*+1^ = *UP* ⊕ *PID*^*x*^ and stores both *PID*^*x*^ and *PID*^*x*+1^ in its memory to avoid the de-synchronization attack. Moreover, *GW* calculates $W_{2}=H(TS_{SN_{x}}∥PID_{SN_{x}}∥T_{y})$, *G*_2_ = *W*_1_ ⊕ *W*_2_, *G*_3_ = (*R*_2_, *R*_1_) ⊕ *W*_2_, and $Auth_{a3}=H(W_{1}∥R_{2}∥R_{1}∥TS_{SN_{x}}∥PID_{SN_{x}}∥T_{y})$. Finally, *GW* creates a message *ME*_*b*_: { *T*_*y*_, *G*_2_, *G*_3_, *Auth*_*a*3_} and sends it to *SN*_*x*_ via the public channel.

#### 3.3.5 Step RAP-5

After procuring *ME*_*b*_ from *GW*, *SN*_*x*_ verifies the condition *TD*_*x*_ ≥ |*Tr* − *T*_*y*_|. If the condition holds, *SN*_*x*_ computes $W_{3}=H(TS_{SN_{x}}∥PID_{SN_{x}}∥T_{y})$, *W*_1_ = *G*_2_ ⊕ *W*_3_, and (*R*_2_, *R*_1_) = *G*_3_ ⊕ *W*_3_. Moreover, *SN*_*x*_ calculates $Auth_{a4}=H(W_{1}∥R_{2}∥R_{1}∥TS_{SN_{x}}∥PID_{SN_{x}}∥T_{y})$. Furthermore, *SN*_*x*_ determines the integrity of *ME*_*b*_ by validating the condition *Auth*_*a*3_ = *Auth*_*a*4_. If the condition holds, *SN*_*x*_ picks a timestamp *T*_*z*_ and a random number *R*_2_, and computes *G*_4_ = *H*(*R*_1_ ∥ *R*_2_ ∥ *R*_3_) ⊕ *W*_1_. For securing communication with *RU*_*y*_, *SN*_*x*_ calculates $SK_{x}=H(H(R_{1}∥R_{2}∥R_{3})∥W_{1}∥T_{z}∥PID_{SN_{x}})$. In addition, *SN*_*x*_ computes *Auth*_*a*5_ = *H*(*H*(*R*_1_ ∥ *R*_2_ ∥ *R*_3_) ∥ *R*_1_ ∥ *T*_*z*_ ∥ *SK*_*x*_). Finally, *SN*_*x*_ calculates a message *ME*_*c*_: {*T*_*z*_, *G*_4_, *Auth*_*a*5_} and sends it to *RU*_*y*_ via the public channel.

#### 3.3.6 Step RAP-6

*RU*_*y*_ considers the received *ME*_*c*_ fresh if the condition *TD*_*z*_ ≥ |*Tr* − *T*_*z*_| holds. If *ME*_*c*_ is valid, *RU*_*y*_ calculates $W_{4}=H(R_{1}∥TS_{RU_{y}}∥PID^{x})$, and *H*(*R*_1_ ∥ *R*_2_ ∥ *R*_3_) = *G*_4_ *W*_4_. For encrypted communication with *SN*_*x*_, *RU*_*y*_ computes $SK_{y}=H(H(R_{1}∥R_{2}∥R_{3})∥W_{4}∥T_{z}∥PID_{SN_{x}})$. Furthermore, *RU*_*y*_ computes *Auth*_*a*6_ = *H*(*H*(*R*_1_ ∥ *R*_2_ ∥ *R*_3_) ∥ *R*_1_ ∥ *T*_*z*_ ∥ *SK*_*y*_) and checks *Auth*_*a*5_ = *Auth*_*a*6_. If the equation holds, *RU*_*y*_ considers *ME*_*c*_ as a valid message. Finally, *RU*_*y*_ computes $UP_{}=W_{4}^{a}⊕W_{4}^{b}$ and updates *PID*^*x*^ by calculating *PID*^*x*+1^ = *PID*^*x*^ ⊕ *UP*_1_. *RU*_*y*_ keeps both *PID*^*x*+1^ and *PID*^*x*^ in its memory to ensure resistance against de-synchronization attack. The user AKE phase of SRUA-IoT is summarized in S1 Fig.

### 3.4 Password change phase

In SRUA-IoT, an authorized user *RU*_*y*_ can change its password and update bio-metric information without involving RC. *RU*_*y*_ needs to perform the following steps to execute the password change phase (PCP).

#### 3.4.1 Step PCP-1

*RU*_*y*_ provides its secret credentials, namely, $ID_{RU_{y}}^{o}$, $PS_{RU_{y}}^{o}$, and $B_{RU_{y}}^{o}$ as inputs at the interface of *SD*_*y*_. After procuring the inputs, *SD*_*y*_ computes the bio-metric key $\beta_{k}^{o}=Rep(B_{RU_{y}}^{o},Rp^{o})$. Moreover, *SD*_*y*_ derives the decryption Key $DK_{lo}^{o}$ by computing $Z_{y}^{o}=H(ID_{RU_{y}}^{o}∥PS_{RU_{y}}^{o}∥\beta_{k}^{o})$, and $DK_{lo}^{o}=(Z_{y}^{o}∥{\left[Z_{y}^{o}\right]}^{32})$. By using AES-192 decryption algorithm, *SD*_*y*_ calculates $PT_{lo}^{o}=D_{DK_{lo}^{o}}\left\{CT_{lo}^{o}\right\}$, where $PT_{lo}^{o}=\{PID^{x},TS_{RU_{y}},PID_{SN_{x}}\}$. Furthermore, *SD*_*y*_ computes $Z_{x}^{o}=H(PID^{x}∥TS_{RU_{y}}∥PID_{SN_{x}}),Auth_{lo}^{o}=H(ID_{RU_{y}}^{o}∥PS_{RU_{y}}^{o}∥\beta_{k}^{o}∥Z_{x}^{o})$, and verifies if the condition $Auth_{lo}^{o}\overset{}{=}Auth_{lo}$ holds. If it holds, *SD*_*y*_ notifies *RU*_*y*_ to enter a new password $PS_{RU_{y}}^{n}$ and update bio-metric information $B_{RU_{y}}^{n}$. Otherwise, *SD*_*y*_ halts the AKE process.

#### 3.4.2 Step PCP-2

Upon procuring $PS_{RU_{y}}^{n}$ and $B_{RU_{y}}^{n}$ from *RU*_*y*_, *SD*_*y*_ determines a new bio-metric key *β*^*n*^ by computing $\left(\beta^{n},Rp^{n})=Gen(B_{RU_{y}}^{n}\right)$. Moreover, *SD*_*y*_ computes the encryption key $EK_{lo}^{n}$ as $Z_{y}^{n}=H(ID_{RU_{y}}^{o}∥PS_{RU_{y}}^{n}∥\beta_{k}^{n})$, $EK_{lo}^{n}=(Z_{y}^{n}∥{\left[Z_{y}^{n}\right]}^{32})$, where ${\left[Z_{y}^{n}\right]}^{32}$ are the first 32 bits of $Z_{y}^{n}$. Furthermore, *SD*_*y*_ calculates new plaintext $PT_{lo}^{n}$ by deriving $PT_{lo}^{n}=\{PID^{x},TS_{RU_{y}},PID_{SN_{x}}\}$. In addition, *SD*_*y*_ computes $Z_{x}^{n}=H(PID^{x}∥TS_{RU_{y}}∥PID_{SN_{x}})$, and $Auth_{lo}^{n}=H(ID_{RU_{y}}^{o}∥PS_{RU_{y}}^{n}∥\beta_{k}^{n}∥Z_{x}^{n})$. Finally, by utilizing AES-192 encryption algorithm, *SD*_*y*_ calculates $CT_{lo}^{n}=E_{EK_{lo}^{n}}\{PT_{lo}^{n}\}$, replaces $\left\{CT_{lo}^{n},Auth_{y}^{n},Rp^{n},Gen(.),Rep(.),Et^{n}\right\}$ with {*CT*_*lo*_, *Auth*_*y*_, *Rp*, *Gen*(.), *Rep*(.), *Et*} in *SD*_*y*_’s memory, and deletes all other credentials in its memory. S2 Fig summarizes PCP.

### 3.5 Revocation phase

If a legitimate *RU*_*y*_ loses its *SD*_*y*_, *RU*_*y*_ can obtain a new $SD_{y}^{new}$ from RC. To obtain $SD_{y}^{new}$, it is necessary for *RU*_*y*_ to remember its $ID_{RU_{y}}$. For proper RP, it is necessary to remove the previous data from *GW*’s memory. Most AKE schemes do not delete the old data from the memory of *GW* or server. *RU*_*y*_ needs to perform the succeeding steps to procure a new SC.

#### 3.5.1 Step RP-1

Upon getting $ID_{RU_{y}}$, *SD*_*y*_ computes $HID_{y}=H(ID_{RU_{y}})$, constructs a message $ME_{1}^{rov}:\left\{HID_{y}\right\}$, and forwards $ME_{1}^{rov}$ to RC. After getting $ME_{1}^{rov}$ from *RU*_*y*_, RC computes *B* = *H*(*GK* ∥ *ID*_*G*_) ⊕ *HID*_*y*_, $RP_{RU_{y}}^{}=B_{}^{a}⊕B_{}^{b}$, and verifies if $RP_{RU_{y}}^{}$ exists in its memory. If found, RC removes $RP_{RU_{y}}^{}$ related record and informs *RU*_*y*_ for new registration by sending $ME_{1}^{rov}:\left\{registration\;\;request\right\}$ to *RU*_*y*_.

#### 3.5.2 Step RP-2

Upon getting the new registration request, *RU*_*y*_ picks new $PS_{RU_{y}}^{new}$, $ID_{RU_{y}}^{new}$, and computes $HID^{new}=H\left(ID_{RU_{y}}^{new}\right)$. *SD*_*y*_ constructs a message $ME_{3}^{rov}:\left\{HID_{y}^{new}\right\}$ and sends to RC.

#### 3.5.3 Step RP-3

RC picks a new pseudonym $PID_{new}^{x}$ for *RU*_*y*_ and computes $A_{q}^{new}=H(GK∥ID_{G})$. To issue a new $SD_{y}^{new}$ to *RU*_*y*_, RC computes the same computation as accomplished in Step RUR-2 of Section 3.2. Finally, RC contrives a message $ME_{4}^{rov}:\{PID_{new}^{x},TS_{RU_{y}}^{new},PID_{SN_{x}}^{new}\}$ and sends $ME_{4}^{rov}$ to *RU*_*y*_ via a reliable channel.

#### 3.5.4 Step RP-4

After receiving $ME_{4}^{rov}$ from RC, *SD*_*y*_ executes the same computation as excuted in Step RUR-3 of Section 3.2 Finally, *SD*_*y*_ stores a new list of parameters {*CT*^*new*^, $Auth_{y}^{new}$, *Gen*(.), *Rep*(.), *Rp*^*new*^, *Et*^*new*^} in *SD*_*y*_’s memory. Moreover, RC stores a list of credentials {$PID_{new}^{x}$, $RP_{RU_{y}}^{new}$, $CT_{RU_{y}}^{new}$} in *GW*’s memory. The revocation phase is summarized in S3 Fig.

### 3.6 New SN deployment phase

RC can deploy a new SN (NSN) by performing the following steps.

#### 3.6.1 Step NSN-1

RC picks a distinct $ID_{SN_{x}}^{n}$ and $PID_{SN_{x}}^{n}$ for NSN $SN_{x}^{n}$. In addition, RC picks $R_{x}^{n}$ and computes a new temporary secret $TS_{SN_{x}}^{n}$ for $SN_{x}^{n}$ by calculating $A_{e}^{n}=H(GK∥R_{x}^{n}∥ID_{G})$, and $TS_{SN_{x}}^{n}=A_{e}^{n-a}⊕A_{e}^{n-b}$, where $A_{e}^{n-a}$ and $A_{e}^{n-b}$ are two chunks of $A_{e}^{n}$, each of size 80 bits.

#### 3.6.2 Step NSN-2

Finally, RC stores the credentials {$ID_{SN_{x}}^{n}$, $PID_{SN_{x}}^{n}$, $TS_{SN_{x}}^{n}$} in $SN_{x}^{n}$’s memory before its deployment.

## 4 Security analysis

In this section informal security analysis of SRUA-IoT is carried out to shows its resistance against various security attacks. The security of SK is validated by utilizing the well-known ROM. Scyther based security analysis is performed to validate SRUA-IoT’s resistance against replay and MITM attacks.

### 4.1 Informal security analysis

This subsection illustrates that the proposed scheme is protected against various attacks, namely, replay, MITM, UI, offline PG, PI, and impersonation attacks.

**Proposition 1** *SRUA-IoT is resistant to replay attack*.

**proof 4.1** *There are three messages exchanged during the execution of the AKE phase, namely, ME_a_: {T_x_, PID^x^, G_1_, Auth_a1_}, ME_b_: {T_y_, G_2_, G_3_, Auth_a3_}, and ME_c_: {T_z_, G_4_, Auth_a5_}. These messages are constructed by incorporating latest timestamps T_x_, T_y_, and T_z_. The freshness of each timestamp is verified by validating the conditions TD_x_ ≥ |Tr − T_x_|, TD_x_ ≥ |Tr − T_y_|, and TD_x_ ≥ |Tr − T_z_| for each message ME_a_, ME_b_, and ME_c_, respectively. If these conditions do not hold, GW, SN_x_, and RU_y_ will detect the replay attack and the receiving network entity will discard the received message. Therefore, SRUA-IoT is resistant to replay attack*.

**Proposition 2** *SRUA-IoT is protected against DoS attack*.

**proof 4.2** *In SRUA-IoT, RU_y_ uses its secret credentials to pass the local authentication, for which SD_y_ needs to calculate* $Auth_{lo}=H(ID_{RU_{y}}∥PS_{RU_{y}}∥\beta_{k}∥Z_{x})$ *and check the condition Auth_y_ = Auth_lo_*. *Local verification will be successful if the condition holds. After local verification, SD_y_ sends the AKE request to GW. Otherwise, SD_y_ terminates the AKE process and prevents RU_y_ from sending a large number of AKE requests to GW. Hence, SRUA-IoT is protected against DoS attack*.

**Proposition 3** *SRUA-IoT ensures untraceability and anonymity of RU*_*y*_.

**proof 4.3** *In SRUA-IoT, during the registration and the AKE phase, only pseudo identities are used, which do not provide any information about* $ID_{RU_{y}}$ . *For each new AKE session, RU*_*y*_ *utilizes the updated PID*^*x*+1^, *and fresh random numbers R*_1_, *R*_2_, *and R*_3_. *During the AKE process, the communicated messages are different for each session. Therefore*, A *cannot correlate the captured message from two different AKE sessions. Thus, SRUA-IoT renders the anonymity and untraceability of RU*_*x*_ *and SN*_*x*_.

**Proposition 4** *SRUA-IoT is protected against MITM attack*.

**proof 4.4** *In SRUA-IoT, there are three messages exchanged, i.e*., *ME*_*a*_, *ME*_*b*_, *and ME*_*c*_. *Let* A *captures the the message ME*_*a*_: {*T*_*x*_, *PID*^*x*^, *G*_1_, *Auth*_*a*1_}, *which is transmitted by RU*_*y*_, *and tries to update the message content by selecting a random number* $R_{1}^{a}$ *and timestamp* $T_{x}^{a}$. *For this*, A *needs to compute* $G_{1}^{a}$ *and* $Auth_{a1}^{a}$ *to pretend that* $ME_{a}^{a}$ *is from a legitimate RU*_*y*_. *However*, A *cannot compute valid* $G_{1}^{}$ *and* $Auth_{a1}^{}$ *without knowing the secret credentials, namely*, $TS_{RU_{y}}$, *and* $PIS_{SN_{x}}$, *which are known only to RU*_*y*_. *We can illustrate the same conditions for ME*_*b*_, *and ME*_*c*_. *Hence, SRUA-IoT is protected against MITM attack*.

**Proposition 5** *SRUA-IoT is immune to offline PG and SSC attacks*.

**proof 4.5** *In this case*, A *can execute various attacks by procuring sensitive information stored on the stolen/lost smart card or device. Let* A *obtains lost/stolen SD*_*y*_ *of RU*_*y*_ *and, by using power analysis attack, can procure the information, such as* {*CT*_*lo*_, *Auth*_*y*_, *Rp*, *Gen*(.), *Rep*(.), *Et*} *stored in the memory of SD*_*y*_. *From the obtained information*, A *cannot retrieve secret credentials, which are used during the AKE process. Therefore, SRUA-IoT is protected against SSC attack. To update the password of RU*_*y*_, A *picks a random identity, password and bio-metric information to compute* $\beta_{k}^{a}=Rep(B_{RU_{y}}^{a},Rp)$, $Z_{y}^{a}=H(ID_{RU_{y}}^{a}∥PS_{RU_{y}}^{a}∥\beta_{k}^{a})$, $DK_{lo}^{a}=\left(Z^{a}y∥{\left[Z_{y}^{a}\right]}^{32}\right)$, *and* $PT_{lo}^{a}=D_{DK_{lo}^{a}}\left\{CT_{lo}\right\}$, *retrieve* $PT_{lo}^{a}=\{PID^{x},PID_{SN_{x}}^{a},TS_{RU_{y}}^{a}\}$, *calculate* $Z_{x}^{a}=H(PID^{x}∥TS_{RU_{y}}^{a}∥PID_{SN_{x}}^{a}),Auth_{a}=H(ID_{RU_{y}}^{a}∥PS_{RU_{y}}^{a}∥\beta_{k}^{a}∥Z_{x}^{a})$, *and check* $Auth_{y}^{a}\overset{}{=}Auth_{lo}$. *However, without knowing the secret credentials of RU*_*y*_, *such as* $ID_{RU_{y}}$, $PS_{RU_{y}}$, *and* $B_{RU_{y}}$, *it is not possible for* A *to perform valid commutation as mentioned above. Therefore, SRUA-IoT is immune to offline PG attack*.

**Proposition 6** *SRUA-IoT is secure against impersonation attack*.

**proof 4.6** *SRUA-IoT considers the following three types of impersonation attacks*.

1. *UI attack: Let* A *tries to generate an AKE request message* $ME_{a}^{a}:\{T_{x}^{a},PID^{x},G_{1}^{a},A^{a}uth_{a1}\}$ *by selecting* $T_{x}^{a}$, *and R*_1_. *However, to send an AKE request to RC*, A *needs to known both the secret credentials, i.e*., $TS_{RU_{y}}$ *and* $PID_{SN_{x}}$, *which are known only to RU*_*y*_. *Moreover*, $TS_{RU_{y}}$ *and* $PID_{SN_{x}}$ *are stored in SD*_*y*_’*s memory in the encrypted form. Therefore, SRUA-IoT is secure against UI attack*.
2. *RC impersonation attack: In this case*, A *picks* $R_{2}^{a}$, $T_{y}^{a}$, *and contrives a message* $ME_{b}^{a}:\{T_{y}^{a},G_{2}^{a},G_{3}^{a},Auth_{a3}^{a}$} *to pretend that this messages is from a legitimate RC. However, to generate* $ME_{b}^{a}$, A *needs to know the secret parameters, such as* $TS_{SN_{x}}$ *and* $PID_{SN_{x}}$, *which are stored in encrypted form. Therefore, without knowing these parameters*, A *cannot fabricate a false massage to make SN*_*x*_ *believe that the message is created by a legal RC. Hence, SRUA-IoT is secure against RC impersonation attack*.
3. *SN*_*x*_ *impersonation attack*: A *can generate a fake message* $ME_{c}^{a}:\{T_{z}^{a},G_{4}^{a},Auth_{a5}^{a}\}$ *and send it to RU*_*y*_ *to make RU*_*y*_ *believe that the message is from a legal SN*_*x*_. *However, to generate a valid* $ME_{c}^{}$, A *needs to know W*_1_, *R*_1_, *R*_2_, *R*_3_, *and* $TS_{SN_{x}}$. *Without the knowledge of these secret credentials, it is impractical for* A *to create a licit message* $ME_{c}^{}$. *Hence, SRUA-IoT is secure against SN*_*x*_ *impersonation attack*.

**Proposition 7** *SRUA-IoT is resilient against SN*_*x*_ *capture attack*.

**proof 4.7** *In 6LoWPANs, SN*_*x*_ *are deployed in unattended environment*. A *can capture an SN*_*x*_ *and can procure the sensitive information stored in the memory of SN*_*x*_. *Since all the deployed SN*_*x*_ *contain distinct secret information, therefore, by capturing an SN*_*x*_ A *cannot breach the security of the entire 6LoWPAN. Hence, SRUA-IoT is resilient against SN*_*x*_ *capture attack*.

**Proposition 8** *SRUA-IoT is immune to de-synchronization attack*.

**proof 4.8** *If the network entities are updating pseudonyms during the execution of the AKE process*, A *can establish de-synchronization attack by dropping the captured message. In SRUA-IoT, GW and RU*_*y*_ *update PID*^*x*^ *to PID*^*x*+1^ *to accomplish anonymous communication. However, to avoid the de-synchronization attack, both GW and RU*_*y*_ *keep PID*^*x*^ *and PID*^*x*+1^ *in their memory. If* A *halts the AKE process by dropping the authentication messages, RU*_*y*_ *can use old PID*^*x*^ *for the AKE process. Therefore, SRUA-IoT is immune to de-synchronization attack*.

**Proposition 9** *SRUA-IoT is resistant to ESL attack*.

**proof 4.9** *Proof In SRUA-IoT, both RU*_*y*_ *and SN*_*x*_ *compute SK as* $SK_{x,y}=H(H(R_{1}∥R_{2}∥R_{3})∥H(R_{1}∥TS_{RU_{y}}∥PID^{x})∥T_{z}∥PID_{SN_{x}})$. *It is obvious that the calculated SK is the concoction of ephemeral (short term) parameters R*_1_, *R*_2_ *and R*_3_, *and long term credential*, $TS_{RU_{y}}$, $PID_{SN_{x}}$, *and PID*^*x*^. A *needs to compromise both ephemeral and long term credentials to reveal SK. Therefore, SRUA-IoT is resistant to ESL attack*.

**Proposition 10** *SRUA-IoT ensures PFS*.

**proof 4.10** *From the discussion in Proposition 9, it is clear that SK is the concatenation of fresh ephemeral and long term secret credentials. If* A *compromises SK of the previous AKE process but cannot compromise SK of the new AKE processes, then SRUA-IoT renders the PFS feature*.

**Proposition 11** *SRUA-IoT ensures secure MA*.

**proof 4.11** *In SRUA-IoT, RU*_*y*_ *achieves validation on RC after verifying the condition* *Auth*_*a*1_ = *Auth*_*a*2_. *For this condition to hold, the knowledge of credentials GK*, *ID*_*G*_, *and* $TS_{RU_{y}}$ *is required. To verify the condition at SN*_*x*_ *Auth*_*a*3_ = *Auth*_*a*4_, *the knowledge of* $TS_{SN_{x}}$ *and* $PID_{SN_{x}}$ *is necessary. SN*_*x*_ *achieves authentication on* $SD_{RU_{y}}$ *by validating the condition* *Auth*_*a*5_ = *Auth*_*a*6_. *Therefore, RU*_*y*_, *SN*_*x*_, *and GW mutually validate each other to achieve secure mutual authentication*.

### 4.2 SK security validation using random oracle model

We employ ROM to corroborate SK’s security in SRUA-IoT. In ROM, A consociates with *k*th instance of a participating entity *EN*^*k*^, which is involved in executing SRUA-IoT. It can be a legitimate *RU*_*y*_, *GW* or *SN*_*x*_. Therefore, $EN_{RU_{y}}^{k}$, $EN_{GW}^{k}$, and $EN_{SN_{x}}^{k}$ are $k_{1}^{th}$, $k_{2}^{th}$, and $k_{3}^{th}$ instances of *RU*_*y*_, *GW*, and *SN*_*x*_, respectively. To simulate real attacks, ROM considers various queries, namely, *Send*, *Test*, *Reveal*, *CorruptSD*, and *Execute*. A description of these queries is presented in S3 Table. Furthermore, SHA is modeled as a random oracle HR (|*HR*| specifies the rage space of SHA output) and it is available for all SRUA-IoT executing entities including A. By using the queries presented in S3 Table, the security of SK is proved in Theorem 4.12.

**Theorem 4.12** *Suppose a polynomial-time* A *is running against the proposed SRUA-IoT in time T*_*i*_. *If QR_h_ denotes the hash quires*, |*HR*| *specifies the range space of SHA output, SQ*_*s*_ *indicates the send queries, lbk defines the length of β*_*k*_ *key, and* |*PD*| *refers to the password dictionary, the approximated advantage of* A *in breaching the security of SRUA-IoT for procuring SK between RU*_*y*_ *and SN*_*x*_ *can be defined as*

$$\begin{array}{c} AD_{A}^{SRUA-IoT}\left(T_{i}\right)\leq \frac{QR_{h}^{2}}{\left|HR\right|}+\frac{SQ_{s}}{2^{lbk-1}\left|PD\right|}. \end{array}$$ (1)

**proof 4.13** *To prove this theorem, we consider the following four games* (*GM*_*x*_|*x* = 0, 1, 2, 3).

#### 4.2.1 *GM*_0_

*A real security attack is accomplished by* A *against SRUA-IoT in GM*_0_. A *picks c bits at GM*_0_. *Therefore, we can procure*

$$\begin{array}{c} AD_{SRUA-IoT}^{A}\left(T_{i}\right)=\left|2.AD_{SRUA-IoT}^{A,GM_{0}}-1\right|. \end{array}$$ (2)

#### 4.2.2 *GM*_1_

*In GM*_1_, A *effectuates an eavesdropping attack and captures all the exchanged messages ME*_*a*_:{*T*_*x*_, *PID*^*x*^, *G*_1_, *Auth*_*a*1_}, *ME*_*b*_:{*T*_*y*_, *G*_2_, *G*_3_, *Auth*_*a*3_}, *and ME*_*c*_:{*T*_*z*_, *G*_4_, *Auth*_*a*5_} *during the AKE process of SRUA-IoT by utilizing the execute query defined in* S3 Table. *To procure SK*, A *executes the Reveal and Test queries and checks if the return key is a random string or real key at the completion of GM*_1_. *The constructed SK between RU*_*y*_ *and SN*_*x*_ *is* $SK_{x,y}=H(H(R_{1}∥R_{2}∥R_{3})∥H(R_{1}∥TS_{RU_{y}}∥PID^{x})∥T_{z}∥PID_{SN_{x}})$. A *needs to know all the long-term secrets and other ephemeral numbers, which are known only to RU*_*y*_, *SN*_*x*_, *and RC. Hence by executing the eavesdropping attack, the chance of* A *to win the game will not be enhanced. Therefore, it is evident that*

$$\begin{array}{c} AD_{SRUA-IoT}^{A,GM_{1}}=AD_{SRUA-IoT}^{A,GM_{0}}. \end{array}$$ (3)

#### 4.2.3 *GM*_2_

*In GM*_2_, A *performs an active attack by simulating Send and Hash quires. All the exchanged messages ME_a_, ME_b_, and ME_c_ are protected using the collision resistance SHA function. The communicated message incorporates random number, timestamps, secret identities, and TSs. Therefore, no SHA collision will occur when* A *effectuates the Send and Hash quarries. By birthday paradox, the following can be achieved*.

$$\begin{array}{c} |AD_{SRUA-IoT}^{A,GM_{1}}-AD_{SRUA-IoT}^{A,GM_{2}}|\leq QR_{h}^{2}/\left(2|HR|\right). \end{array}$$ (4)

#### 4.2.4 *GM*_3_

*This game effectuates the simulation of CorruptSD query. Typically, RU_y_ picks low-entropy passwords. By utilizing the password dictionary attack*, A *tries to guess the password of RU_y_ after procuring the information stored on SD_y_, including {CT*_*lo*_, *Auth_y_, Rp, Gen(.), Rep(.), Et*}. A *also attempts to guess β*_*k*_ *from the information stored on SD_y_. SRUA-IoT employs robust FE that generates highly random β_k_* ∈ [0, 1]^*lbk*^, *where lbk is the length of β_k_. The probability of guessing β_k_ is nearly* $\frac{1}{2^{lbk}}$. *Furthermore, in the communication system, only a limited number of wrong password attempts are allowed. Under these conditions, we have*

$$\begin{array}{c} |AD_{SRUA-IoT}^{A,GM_{2}}-AD_{SRUA-IoT}^{A,GM_{3}}|\leq \frac{SQ_{s}}{2^{lbk}\left|PD\right|}. \end{array}$$ (5)

*After executing the above queries*, A *needs to guess bit c upon executing the Test query. Therefore, we have* $AD_{SRUA-IoT}^{A,GM_{3}}=\frac{1}{2}$.

*By utilizing the triangular inequality and simplifying* (2)–(5), *the following is achieved*:

$$\begin{array}{c} \begin{array}{c} \frac{1}{2}AD_{SRUA-IoT}^{A}\left(T_{i}\right)=\left|AD_{SRUA-IoT}^{A,GM_{3}}=\frac{1}{2}\right|\\ =|AD_{SRUA-IoT}^{A,GM_{1}}-AD_{SRUA-IoT}^{A,GM_{3}}|\\ \leq |AD_{SRUA-IoT}^{A,GM_{1}}-AD_{SRUA-IoT}^{A,GM_{2}}|\\ +|AD_{SRUA-IoT}^{A,GM_{2}}-AD_{SRUA-IoT}^{A,GM_{3}}|\\ \leq \frac{QR_{h}^{2}}{2|HR|}+\frac{SQ_{s}}{2^{lbk}\left|PD\right|}.\;\;\;\;\;\;\;\;\;\;\;\;\;\;\; \end{array} \end{array}$$ (6)

*Hence, we get*

$$\begin{array}{c} AD_{SRUA-IoT}^{A}\left(T_{i}\right)\leq \frac{QR_{h}^{2}}{\left|HR\right|}+\frac{SQ_{s}}{2^{lbk-1}\left|PD\right|}. \end{array}$$ (7)

### 4.3 Scyther analysis

We employ the well-known formal security validation tool, called Scyther [68], to validate the security properties and correctness of the proposed SRUA-IoT scheme. To that end, the security protocol description language (SPDL) is utilized to specify SRUA-IoT by employing the operational semantics ascertained in [68]. S4 Fig demonstrates that proclaims are satisfied, which are specified in the SPDL script. In S4 Fig, SRUA-IoT is the name of the devised protocol with the initiator *RU* and *RC* as the helper node and *SN* as the responder. The descriptions of Nisynch and secrecy are provided in [68]. Secrecy signifies that specific information is not disclosed to any attacker, even when the information is exchanged over a public network. Furthermore, Nisynch describes that any claim defined in the devised protocol specification will also appear in the trace. Moreover, SRUA-IoT analysis illustrates that the supplementary security characteristics produced by Scyther, namely, weak agreement (Weakagree), aliveness (Alive), and non-injective agreement (Niagree) are validated.

## 5 Performance evaluation

In this section, the performance of SRUA-IoT is compared with Park *et al*. [69], Shuai *et al*. [36], Das *et al*. [30], Shin *et al*. [31], Challa *et al*. [22], Srinivas *et al*. [33], Wazid *et al*. [35], and Chen *et al*. [27] in terms of computational cost, communication cost, security features, and storage cost. We use *C*/*C*^++^ based cryptographic library MIRACL and Raspberry PI-3 (RPI-3B) with Quad-core @1.2 GHz, 1BG of RAM, and Ubuntu 16.04 LTS for implementing the proposed SRUA-IoT and the relevant AKE schemes.

### 5.1 Security features

The proposed SRUA-IoT is compared with the relevant AKE scheme in terms of security functionalities and resistance against various attacks. S4 Table exhibits that Park *et al*. [69] is unprotected against UA, SSC, and PT attacks, Shuai *et al*. [36] is unsafe against de-synchronization attacks, Das *et al*. [30] cannot withstand SSC, PI, and UA attacks and does not ensure SK security, Shin *et al*. [31] is insecure against de-synchronization attack and does not provide revocation phase, Challa *et al*. [22] cannot withstand PI, SSC, UA, PG, and UI attacks, Srinivas *et al*. [33] fails to protect against UI, PI, and SSC attacks, Wazid *et al*. [35] is unsafe against UI, PI, and SSC attacks, and Chen *et al*. [27] cannot protect PI, PG, UA, UI, replay and DoS attacks and also does not ensure mutual authentication. Contrarily, SRUA-IoT is secure as compared to the relevant eminent AKE schemes, as shown in S4 Table.

### 5.2 Computational cost

In this subsection, the approximated computational overhead of SRUA-IoT and relevant AKE schemes is determined by using computational time of various cryptographic primitives presented in S5 Table. SRUA-IoT has a computational cost of 19*T*_*SA*_ + 2*T*_*ED*_ + $T_{\beta_{k}}\approx 6.901$ ms, which is less than the benchmark schemes, as shown in S5 Fig and S6 Table. SRUA-IoT has 53.09%, 23.88%, 44.23%, 29.56%, 22.04%, 76.41%, 24.07%, and 38.93% less computational cost as compared to Park *et al*. [69], Shuai *et al*. [36], Das *et al*. [30], Shin *et al*. [31], Srinivas *et al*. [33], Challa *et al*. [22], Wazid *et al*. [35], and Chen *et al*. [27], respectively. Furthermore, SRUA-IoT has a computational overhead of 5*T*_*SA*_ ≈ 1.275ms at *SN*_*x*_, which is less than the benchmark AKE schemes, as shown in S6 Fig and S6 Table. The computational overhead at *GW* increases with the number of users accessing the network resources. S7 Fig shows that SRUA-IoT requires low computational overhead while processing multiple AKE requests simultaneously.

Although the security of SRUA-IoT is verified through formal and informal analyses in Section 4 where the scheme has been shown to resist various covert security attacks, however, an attack or some unexpected event can halt the execution of SRUA-IoT, which may occur at any step of the AKE phase. Under a specific attack, the execution time can be computed as

$$\begin{array}{c} T_{atp}=\frac{\sum_{i}^{100}T_{i}}{\left(1-\text{attack}\;\text{success}\;\text{probability}\right)}, \end{array}$$ (8)

where *T*_*i*_ denotes time required to accomplish the AKE phase and $\sum_{i}^{100}T_{i}$ denotes the average time, which is procured after running SRUA-IoT 100 times, and *T*_*atp*_ denotes the execution time required to complete the AKE phase under successful attack probability. S8 Fig demonstrates the time utilization of SRUA-IoT and other related schemes with attack success probability. Under various successful attack attempts, SRUA-IoT requires less time to complete its execution than the related AKE schemes.

### 5.3 Communication cost

The comparative analysis of communication cost is illustrated in this subsection. For SRUA-IoT, the size of timestamp is 32 bits, *ECC* point is 160 bits, SHA output size is 160 bits, random number size is 80 bits, different *PID* size is 80 bits, and AES key size is 192 bits. During the execution of the AKE phase, SRUA-IoT exchanges three message, namely, *ME*_*a*_: {*T*_*x*_, *PID*^*x*^, *G*_1_, *Auth*_*a*1_}, *ME*_*b*_: {*T*_*y*_, *G*_2_, *G*_3_, *Auth*_*a*3_ and *ME*_*c*_: {*T*_*z*_, *G*_4_, *Auth*_*a*5_}, of length {32 + 80 + 160 + 160} = 432 bits, {32 + 160 + 160 + 160} = 512 bits, and {32 + 160 + 160} = 412 bits, respectively. The aggregated communication overheads of SRUA-IoT is 1356 bits. S7 Table and S9 Fig demonstrate the comparison of SRUA-IoT and other related AKE schemes. SRUA-IoT has 75.92%, 21.53%, 11.72%, 29.28%, 46.36%, 11.72%, 20.05%, and 57.2% less communication cost as compared to Park *et al*. [69], Shuai *et al*. [36], Das *et al*. [30], Shin *et al*. [31], Challa *et al*. [22], Srinivas *et al*. [33], and Chen *et al*. [27], respectively.

### 5.4 Storage cost

This subsection provides the storage cost comparison of SRUA-IoT with other AKE schemes. In SRUA-IoT, *RU*_*y*_, *GW*, and *SN*_*x*_ store {*CT*_*lo*_, *Auth*_*y*_, *Rp*, *Gen*(.), *Rep*(.), *Et*}, {*PID*^*x*+1^, *PID*^*x*^, $RP_{RU_{y}}$, $CT_{RU_{y}}$}, and {$PID_{SN_{x}}$, $TS_{SN_{x}}$} of length {240 + 160 + 160 + 8} = 568 bits, {80 + 80 + 80 + 240} = 480 bits, and {80 + 80} = 160 bits, respectively. The total storage overhead can be calculated as {568 + 480 + 160} = 1208 bits. Besides, the storage costs of Park *et al*. [69], Shuai *et al*. [36], Das *et al*. [30], Shin *et al*. [31], Challa *et al*. [22], Srinivas *et al*. [33], Wazid *et al*. [35], and Chen *et al*. [27] are 1600 bits, 1776 bits, 3738 bits, 1160 bits, 4016 bits, 2888 bits, 4126 bits, and 1792 bits, respectively. SRUA-IoT has a slightly higher storage cost as compared to Shin *et al*. [31]. However, SRUA-IoT has less computational and communication cost during the AKE phase in contrast to Shin *et al*. [31]. S10 Fig illustrates the storage cost comparison of SRUA-IoT and the related AKE schemes.

## 6 Conclusion

Information security is critical in resource-constricted 6LoWPAN-based IoT networks. This paper has presented an AKE scheme called SRUA-IoT for resource-constricted 6LoWPAN devices to validate the legitimacy of remote users interacting in real-time with sensor nodes deployed in smart home networks. The scheme performs user authorization before procuring real-time data from sensors by employing a lightweight secure hash algorithm (SHA-160) and an advanced encryption standard (AES-192) to accomplish the AKE process. The proposed scheme is corroborated both formally and informally to explicate its resistance against various malicious security vulnerabilities. Moreover, numerical results in comparison with benchmarks reveal that SRUA-IoT requires low computational and communication resources in 6LoWPANs to accomplish the AKE phase. Our future work will explore authenticated encryption with associated data to devise a resource-efficient AKE scheme with reduced computational cost for resource-constricted IoT devices.

## Supporting information

S1 Fig The user AKE phase of SRUA-IoT. (TIF)

S2 Fig Password change phase. (TIF)

S3 Fig Revocation phase. (TIF)

S4 Fig Scyther results. (TIF)

S5 Fig Comparison of total computation cost required to complete the AKE process. (TIF)

S6 Fig Computational overhead at *SN*_*x*_ side. (TIF)

S7 Fig Computational delay at *GW* with increasing number of users. (TIF)

S8 Fig Computational overhead with attack success probability. (TIF)

S9 Fig Communication overhead in the network with increasing number of users. (TIF)

S10 Fig Comparison of storage costs. (TIF)

S1 Table Comparative analysis of eminent AKE schemes [21–41]. (PDF)

S2 Table List of key notations. (PDF)

S3 Table Description of different ROM queries. (PDF)

S4 Table Comparison of security features [22, 30, 31, 33, 35, 36, 69]. (PDF)

S5 Table Experimental computational cost of various cryptographic operations. (PDF)

S6 Table Comparison of computational costs [22, 27, 30, 31, 33, 35, 36, 69]. (PDF)

S7 Table Comparison of communication costs [22, 27, 30, 31, 33, 35, 36, 69]. (PDF)

S1 Appendix (ZIP)
