# Supplementary figures and images for: A secure remote user authentication scheme for 6LoWPAN-based Internet of Things
Source: PLoS One. 2021 Nov 8;16(11):e0258279. doi: 10.1371/journal.pone.0258279 (PMC8575280; doi:10.1371/journal.pone.0258279)

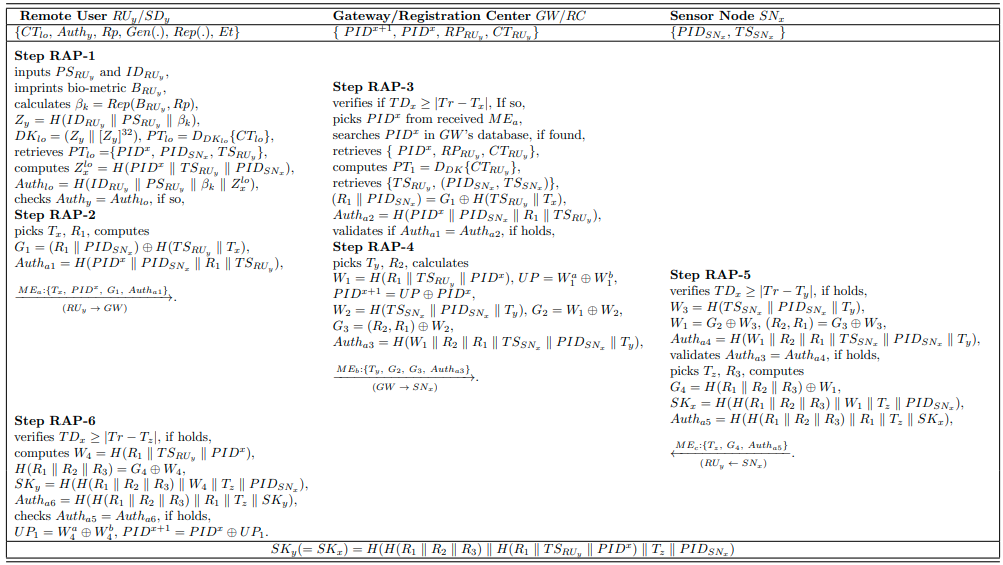

Supplement: S1 Fig — (TIF) [file pone.0258279.s001.tif]

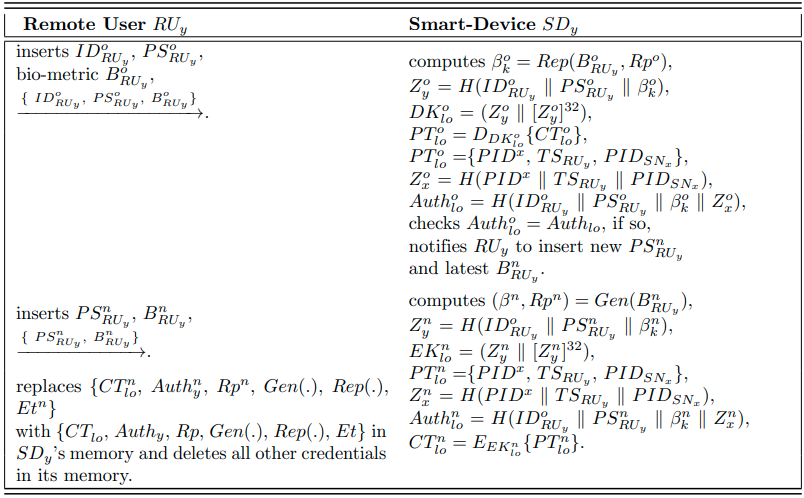

Supplement: S2 Fig — (TIF) [file pone.0258279.s002.tif]

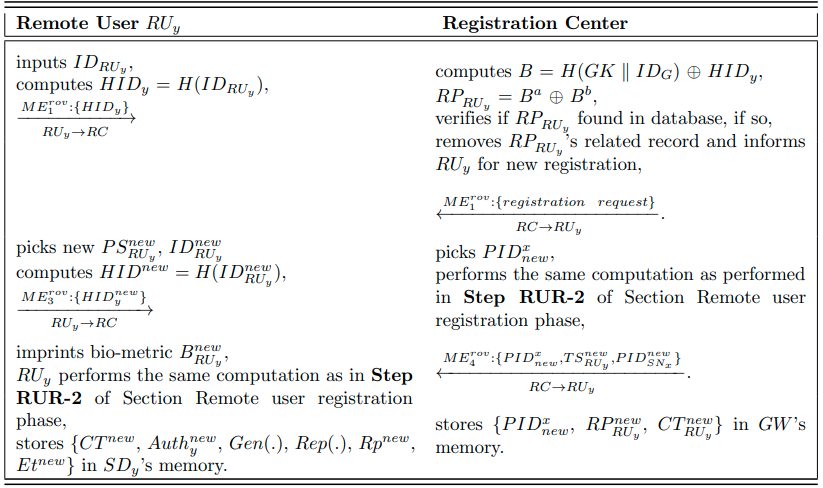

Supplement: S3 Fig — (TIF) [file pone.0258279.s003.tif]

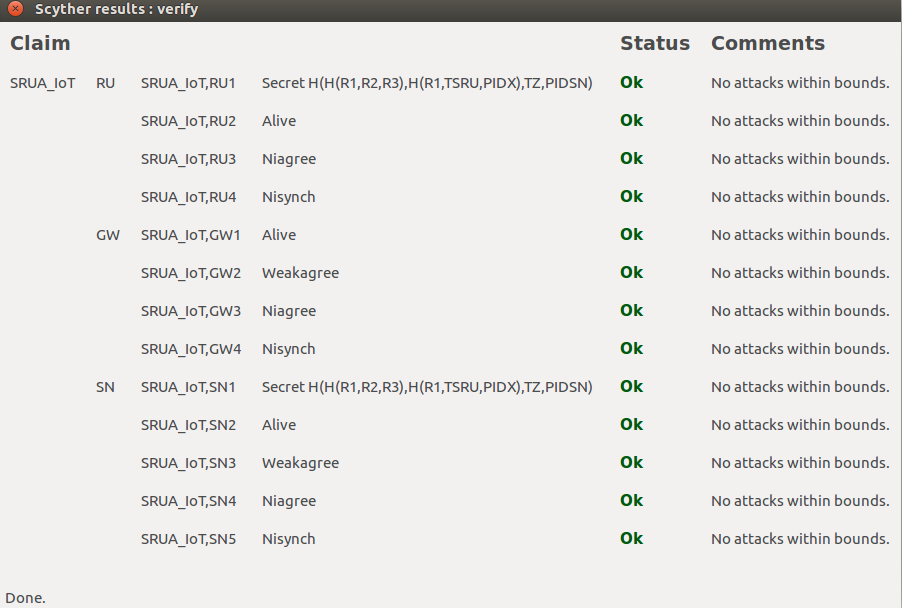

Supplement: S4 Fig — (TIF) [file pone.0258279.s004.tif]

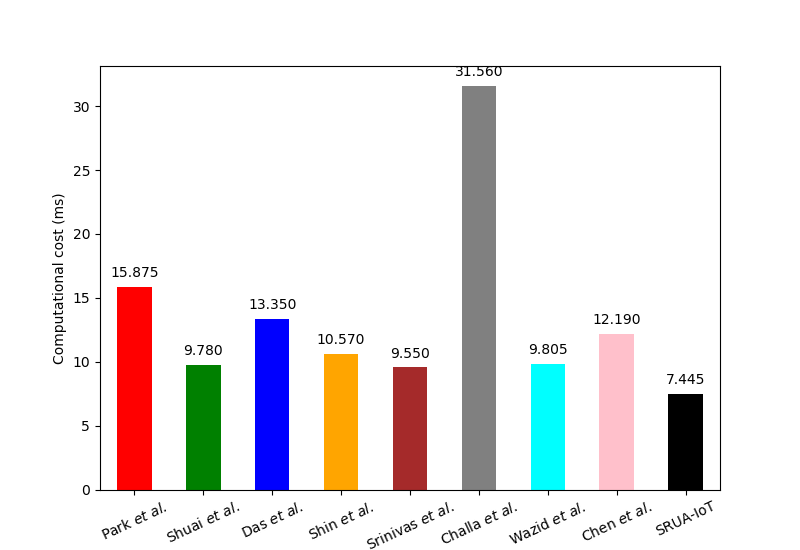

Supplement: S5 Fig — (TIF) [file pone.0258279.s005.tif]

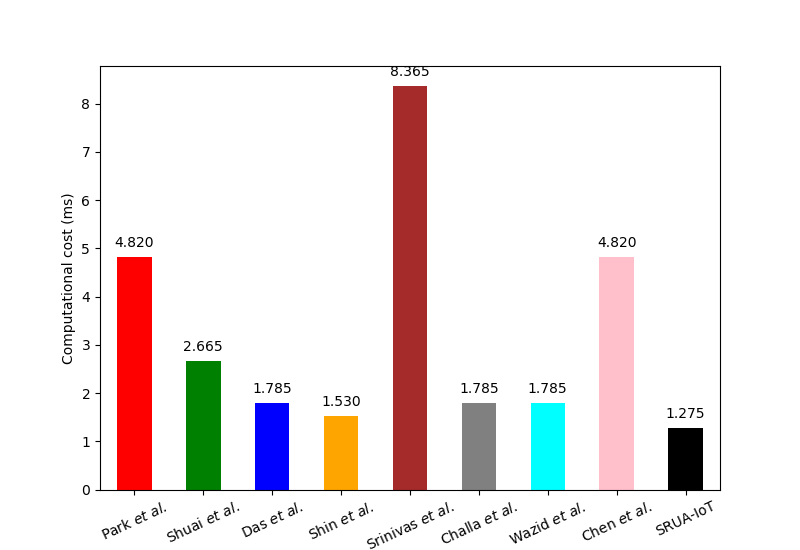

Supplement: S6 Fig — (TIF) [file pone.0258279.s006.tif]

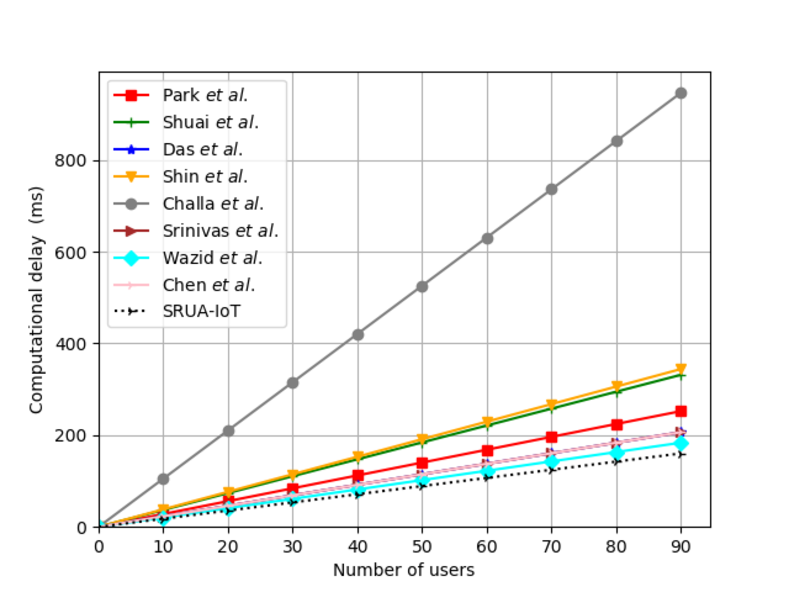

Supplement: S7 Fig — (TIF) [file pone.0258279.s007.tif]

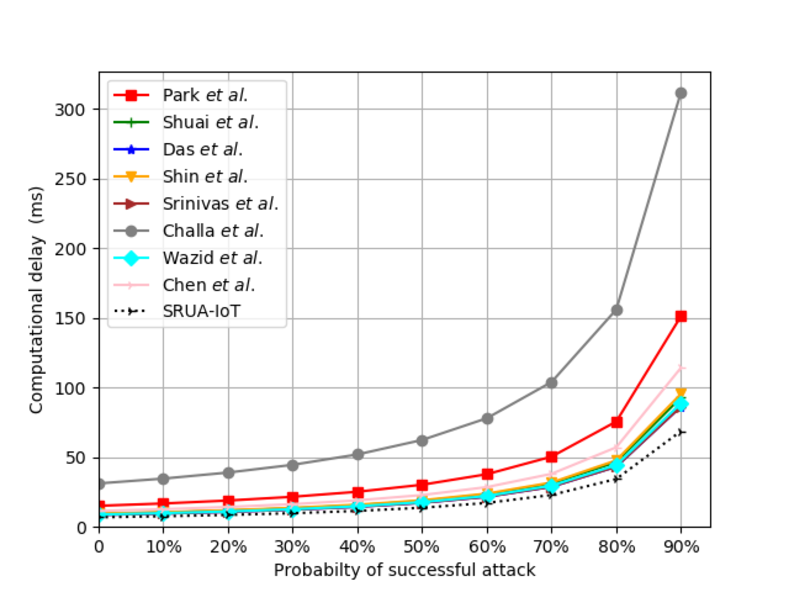

Supplement: S8 Fig — (TIF) [file pone.0258279.s008.tif]

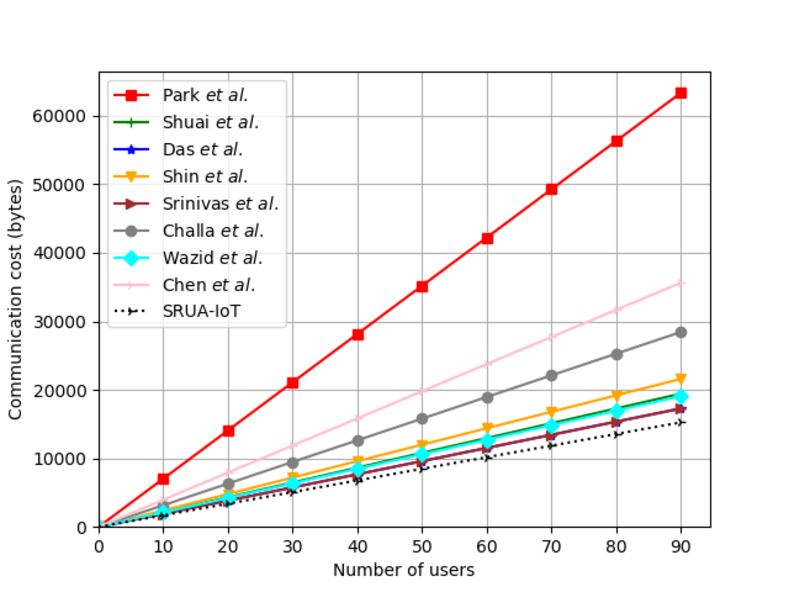

Supplement: S9 Fig — (TIF) [file pone.0258279.s009.tif]

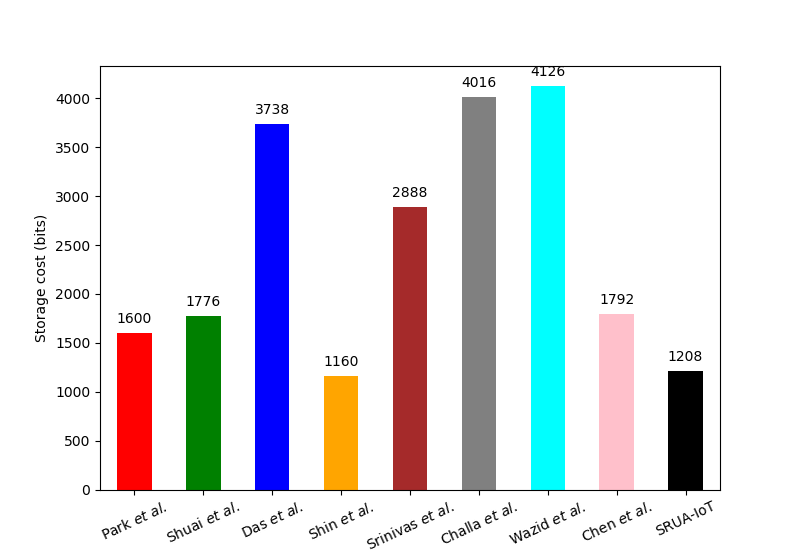

Supplement: S10 Fig — (TIF) [file pone.0258279.s010.tif]
