# Supplementary material for: A secure remote user authentication scheme for 6LoWPAN-based Internet of Things
Source: PLoS One. 2021 Nov 8;16(11):e0258279. doi: 10.1371/journal.pone.0258279 (PMC8575280; doi:10.1371/journal.pone.0258279)
Supplement: S1 Table — (PDF) [file pone.0258279.s011.pdf]

S1 Table Comparative analysis of eminent AKE schemes

AKE scheme	Shortcoming	Year	Operations
Qui and Ma [21]	Unsafe against sinkhole and chosen plaintext attacks	2016	XOR, ECC, and SHA-160
Challa *et al.* [22]	Prone to replay, DoS, forgery and UI attacks	2017	ECC, XOR, and SHA-160
Vijayakumar *et al.* [23]	Cannot resist replay attack and does not provide strong privacy	2017	XOR, AES, and SHA-160
Jung *et al.* [24]	Cannot resist against UI and ESL attacks	2017	XOR and SHA-160
Qi *et al.* [25]	Fragile to PC, PI, UI, UA and offline PG attacks and also does not provide MA	2017	XOR and SHA-160
Chaudhry *et al.* [26]	Vulnerable to offline PG, SSC, UI, and UA attacks	2018	XOR and SHA-160
Chen *et al.* [27]	Does not render MA and anonymity features. Prone to replay, SSD, PI, PG, UI, and DoS attacks	2018	XOR, ECC, and SHA-160
Amin *et al.* [28]	Cannot resist PI, UI, PG, and IG attacks	2018	XOR and SHA-160
Das *et al.* [29]	Does not render the SK's security. Fragile to SSD and traceability attacks	2018	XOR and SHA-160
Das *et al.* [30]	Susceptible to device impersonation and MITM attacks	2018	XOR, ECC, and SHA-160
Shin *et al.* [31]	Unprotected against de-synchronization attack and suffers a design flaw	2019	XOR and SHA-256
Lu *et al.* [32]	Prone to UI and SCC and does not provide a login phase	2019	XOR, ECC, and SHA-160
Srinivas *et al.* [33]	Does not scale well. Susceptible to traceability, UI, and DI attacks	2019	XOR and SHA-160
Mishra *et al.* [34]	Prone to SSD, stolen verifier, UI, and UA attacks and unable to ensure SN's anonymity	2018	XOR, AES, and SHA-160
Wazid *et al.* [35]	Prone to UI, IG, and DI attacks	2018	XOR and SHA-160
Shuai *et al.* [36]	Prone to parallel session, UI, PG, PI, and SSD attacks	2019	XOR, ECC, and SHA-160
Shin *et al.* [31]	Suffers a design flaw and prone to de-synchronization	2019	XOR and SHA-160
Barman *et al.* [37]	Fragile to SI, UI, ESL, SK leakage, and UA attacks	2019	XOR and SHA-160
Singh *et al.* [38]	Susceptible to UA, MITM, UI, SI, and modification attacks, and does not render untraceability, MA, and SK agreement features	2019	XOR and SHA-160
Sadhukhan *et al.* [39]	does not provide password change mechanism. Vulnerable to replay, UA, DoS, and MITM attacks	2021	XOR, ECC, and SHA-160
Ali *et al.* [40]	Unprotected against DoS, forgery, PI, SSD, and SI attacks, and does not render PFS and key freshness features	2020	XOR, AES, and SHA-16
Vinoth *et al.* [34]	Unable to restrain SSD, DoS, replay, and SN capture attacks	2020	XOR, AES, and SHA-160
Tanveer *et al.* [41]	Cannot resist de-synchronization attack	2020	XOR, ASCON, and SHA-256
The proposed scheme (SRUA-IoT)	Can withstand all known attacks. However, the computational cost can be reduced further	2021	XOR, AES-192, and SHA-160

MA: Mutual authentication, PC: Password change, PG: Password guessing, PI: Privileged insider.
