# Supplementary material for: A secure remote user authentication scheme for 6LoWPAN-based Internet of Things
Source: PLoS One. 2021 Nov 8;16(11):e0258279. doi: 10.1371/journal.pone.0258279 (PMC8575280; doi:10.1371/journal.pone.0258279)
Supplement: S2 Table — (PDF) [file pone.0258279.s012.pdf]

S2 Table List of key notations

**Notation**	**Description**
GK, ID_G	Gateway-secret key and identity of gateway, respectively
ID_{RU_y}, PS_{RU_y}	Identity and password of RU_y, respectively
PID^x, PID^{x+1}, TS_{RU_y}	Pseudonyms (old & new) used during AKE and temporal-secret of RU_y
PID_{SN_x}, TS_{SN_x}	Pseudo-identity and temporal-secret of SN_x, respectively
T_x, T_y, T_z	Timestamps used during the AKE phase
$E_{EK}(st)$, $D_{DK}(st)$	Encryption/decryption of string st, respectively
R_1, R_2, R_3	Random number used during the AKE phase
$\|$, $\oplus$, $H(.)$	Concatenation, XOR, and hash-function, respectively
$Gen(.)$, $Rep(.)$, Rp, β_k	FE bio-metric key generation, regeneration, reproduction parameter, and bio-metric key, respectively
B_{RU_y}	Bio-metric information of RU_y
