# Supplementary material for: A secure remote user authentication scheme for 6LoWPAN-based Internet of Things
Source: PLoS One. 2021 Nov 8;16(11):e0258279. doi: 10.1371/journal.pone.0258279 (PMC8575280; doi:10.1371/journal.pone.0258279)
Supplement: S3 Table — (PDF) [file pone.0258279.s013.pdf]

S3 Table Description of different ROM queries

**Query**	**Description**
$Send(EN^k, MSG)$	This query enables $\mathcal{A}$ to launch an active attack by sending a message MSG to EN^t, and EN^t responds accordingly.
$Test(EN^k)$	This query enables $\mathcal{A}$ to make an SK request to EN^k. The response from EN^k is probabilistic like flip coin c.
$Reveal(EN^k)$	This query eables $\mathcal{A}$ to procure an SK, established between EN^t and other partner entities.
$CorruptSD(EN^k_{RU_y})$	By simulating this query, $\mathcal{A}$ can procure RU_y's secret information, namely, PS_{RU_y}, ID_{RU_y}, and β_k.
$Execute(EN^k_{RU_y}, EN^k_{GW}, EN^k_{SN_x})$	This query enables $\mathcal{A}$ to capture all messages exchanged among RU_y, GW, and SN_x.
