# Supplementary material for: A secure remote user authentication scheme for 6LoWPAN-based Internet of Things
Source: PLoS One. 2021 Nov 8;16(11):e0258279. doi: 10.1371/journal.pone.0258279 (PMC8575280; doi:10.1371/journal.pone.0258279)
Supplement: S4 Table — (PDF) [file pone.0258279.s014.pdf]

S4 Table Comparison of security features

Feature	**Park** *et al.* **[69]**	**Shuai** *et al.* **[36]**	**Das** *et al.* **[30]**	**Shin** *et al.* **[31]**	**Challa** *et al.* **[22]**	**Srinivas** *et al.* **[33]**	**Wazid** *et al.* **[35]**	**Chen** *et al.* **[27]**	**SRUA-IoT**
UA	×	✓	×	✓	×	✓	✓	×	✓
UI	✓	✓	✓	✓	×	×	×	×	✓
DS	✓	✓	×	×	✓	✓	✓	✓	✓
SSC/SSD	×	×	✓	✓	×	×	×	✓	✓
PI	×	×	×	✓	×	×	×	×	✓
PG	✓	×	✓	✓	×	✓	✓	×	✓
UT	✓	✓	✓	✓	✓	✓	✓	✓	✓
ESL	✓	✓	×	✓	✓	✓	✓	✓	✓
SKS	✓	✓	×	✓	✓	✓	✓	✓	✓
DoS	✓	✓	×	✓	✓	✓	✓	×	✓
MITM	✓	✓	×	✓	✓	✓	✓	✓	✓
RA	✓	✓	×	✓	✓	✓	✓	×	✓
MA	✓	✓	✓	✓	✓	✓	✓	✓	✓
RP	✓	✓	×	×	✓	✓	✓	✓	✓
NDD	✓	✓	✓	✓	✓	✓	✓	✓	✓
BAN	✓	✓	×	✓	✓	×	×	✓	✓
ROM	✓	✓	✓	✓	✓	✓	×	✓	✓

Note: DS: de-synchronization, RA: replay attack, SKS: SK security, ROM: validation using ROM.
