# Supplementary material for: A secure remote user authentication scheme for 6LoWPAN-based Internet of Things
Source: PLoS One. 2021 Nov 8;16(11):e0258279. doi: 10.1371/journal.pone.0258279 (PMC8575280; doi:10.1371/journal.pone.0258279)
Supplement: S5 Table — (PDF) [file pone.0258279.s015.pdf]

S5 Table Experimental computational cost of various cryptographic operations

Notation	Operation	Computational time
T_{SA}	Hash function	0.255 ms
T_{ED}	AES-192	0.350 ms
T_{ECC}	ECC point multiplication	1.9 ms
$T_{Bk} \approx T_{ECC}$	Fuzzy extractors	1.9 ms
