# Supplementary material for: A secure remote user authentication scheme for 6LoWPAN-based Internet of Things
Source: PLoS One. 2021 Nov 8;16(11):e0258279. doi: 10.1371/journal.pone.0258279 (PMC8575280; doi:10.1371/journal.pone.0258279)
Supplement: S6 Table — (PDF) [file pone.0258279.s016.pdf]

S6 Table Comparison of computational costs

**Scheme**	RU_y **Side**	RC/CS **Side**	D_j/SN_x **Side**	**Total Time**
Park *et al.* [69]	$10T_{SA} + 2T_{EC} + T_{\beta_k}$	$11T_{SA}$	$4T_{SA} + 2T_{EC} \approx 4.82$ ms	$25T_{SA} + 4T_{EC} + T_{\beta_k} \approx 15.87$ ms
Shuai *et al.* [36]	$6T_{SA} + T_{EC}$	$7T_{SA} + T_{EC}$	$3T_{SA} + T_{EC} \approx 2.665$ ms	$16T_{SA} + 3T_{EC} \approx 9.780$ ms
Das *et al.* [30]	$14T_{SA} + 2T_{EC} + T_{\beta_k}$	$9T_{SA}$	$7T_{SA} \approx 1.785$ ms	$30T_{SA} + 2T_{EC} + T_{\beta_k} \approx 13.35$ ms
Shin *et al.* [31]	$13T_{SA} + T_{\beta_k}$	$15T_{SA}$	$6T_{SA} \approx 1.53$ ms	$34T_{SA} + T_{\beta_k} \approx 10.570$ ms
Srinivas *et al.* [33]	$14T_{SA} + T_{\beta_k}$	$9T_{SA}$	$7T_{SA} \approx 1.785$ ms	$30T_{SA} + T_{\beta_k} \approx 9.550$ ms
Challa *et al.* [22]	$5T_{SA} + 5T_{EC} + T_{\beta_k}$	$4T_{SA} + 5T_{EC}$	$3T_{SA} + 4T_{EC} \approx 8.365$ ms	$12T_{SA} + 14T_{EC} + T_{\beta_k} \approx 31.560$ ms
Wazid *et al.* [35]	$16T_{SA} + T_{\beta_k}$	$8T_{SA}$	$7T_{SA} \approx 1.785$ ms	$30T_{SA} + T_{\beta_k} \approx 9.805$ ms
Chen *et al.* [27]	$5T_{SA} + 2T_{EC}$	$9T_{SA}$	$4T_{SA} + 2T_{EC} \approx 4.82$ ms	$18T_{SA} + 4T_{EC} \approx 12.19$ ms
SRUA-IoT	$8T_{SA} + T_{ED} + T_{\beta_k}$	$6T_{SA} + T_{ED}$	$5T_{SA} \approx 1.275$ ms	$19T_{SA} + 2T_{ED} + T_{\beta_k} \approx 7.445$ ms
