# Supplementary material for: A secure remote user authentication scheme for 6LoWPAN-based Internet of Things
Source: PLoS One. 2021 Nov 8;16(11):e0258279. doi: 10.1371/journal.pone.0258279 (PMC8575280; doi:10.1371/journal.pone.0258279)
Supplement: S7 Table — (PDF) [file pone.0258279.s017.pdf]

S7 Table Comparison of communication costs

**Scheme**	**Messages Communicated During AKE Phase**	**Total (bits)**
Park *et al.* [69]	$RU_y \xrightarrow{1536} GW \xrightarrow{1408} SN_x \xrightarrow{1280} GW \xrightarrow{480} RU_y$	5632
Shuai *et al.* [36]	$RU_y \xrightarrow{768} GW \xrightarrow{320} SN_x \xrightarrow{320} GW \xrightarrow{320} RU_y$	1728
Das *et al.* [30]	$RU_y \xrightarrow{672} GW \xrightarrow{512} SN_x \xrightarrow{352} RU_y$	1536
Shin *et al.* [31]	$RU_y \xrightarrow{512} GW \xrightarrow{512} SN_x \xrightarrow{384} GW \xrightarrow{512} RU_y$	1920
Challa *et al.* [22]	$RU_y \xrightarrow{992} GW \xrightarrow{1024} j \xrightarrow{512} RU_y$	2528
Srinivas *et al.* [33]	$RU_y \xrightarrow{672} GW/RC \xrightarrow{512} SN_x \xrightarrow{352} RU_y$	1536
Wazid *et al.* [35]	$RU_y \xrightarrow{672} GW/RC \xrightarrow{512} SN_x \xrightarrow{512} RU_y$	1696
Chen *et al.* [27]	$RU_y \xrightarrow{928} GW/RC \xrightarrow{672} SN_x \xrightarrow{896} GW/RC \xrightarrow{672} RU_y$	3168
SRUA-IoT	$RU_y \xrightarrow{432} GW \xrightarrow{512} SN_x \xrightarrow{412} RU_y$	1356
