# Supplementary material for: A secure remote user authentication scheme for 6LoWPAN-based Internet of Things
Source: PLoS One. 2021 Nov 8;16(11):e0258279. doi: 10.1371/journal.pone.0258279 (PMC8575280; doi:10.1371/journal.pone.0258279)
Supplement: S1 Appendix — (ZIP) [file pone.0258279.s018.zip › Supporting_information.pdf]

Supporting Information

S1 Fig The user AKE phase of SRUA-IoT.
S2 Fig Password change phase.
S3 Fig Revocation phase.
S4 Fig Scyther results.
S5 Fig Comparison of total computation cost required to complete the AKE process.
S6 Fig Computational overhead at SN_x side.
S7 Fig Computational delay at GW with increasing number of users.
S8 Fig Computational overhead with attack success probability.
S9 Fig Communication overhead in the network with increasing number of users.
S10 Fig Comparison of storage costs.
S1 Table Comparative analysis of eminent AKE schemes
S2 Table List of key notations
S3 Table Description of different ROM queries
S4 Table Comparison of security features
S5 Table Experimental computational cost of various cryptographic operations
S6 Table Comparison of computational costs
S7 Table Comparison of communication costs

Appendix

A A Review of Shin *et al.* [1]

This section presents a critical review of the security scheme presented in [1]. We demonstrate that the scheme is insecure against de-synchronization attack. Table 1 tabulates a list of notations used in [1].

A.1 User registration phase

- U_i picks ID_i^s, PW_i^s, and imprints its bio-metric Bio_i^s. U_i selects a random number u_i^s, computes $(b_i^s, par_i^s) = Gen(Bio_i^s)$, $HPW = h(PW_i^s \parallel b_i^s)$, $TID_i^s = h(ID_i^s \parallel u_i^s)$, and dispatches a registration request $\langle TID_i^s, HPW_i^s \rangle$ to HG.

- Upon procuring user's registration message, HG picks one-time pseudonym PID_i^{s1} for U_i and computes $HID_i^s = h(TID_i^s \parallel K_u^s)$, $A_i^s = h(HPW_i^s \parallel TID_i^s) \oplus HID_i^s$, $B_i^s = h(HPW_i^s \parallel HID_i^s)$, and $C_i^{s1} = h(TID_i^s \parallel HID_i^s) \oplus PID_i^{s1}$.

- HG dispatches SC_i to U_i by storing $\{A_i^{s1}, B_i^{s1}, C_i^{s1}\}$ via a secure channel and also stores $\{TID_i, PID_i^{s1}\}$ in its database.

- Finally, U_i computes $D_i^s = u_i^s \oplus h(ID_i^s \parallel b_i^s)$ and stores the parameters $\{A_i^s, B_i^s, C_i^{s1}, D_i^s, par_i^s$, Gen(.), Rep(.)$\}$

Table 1. List of notations used in [1]

Symbol	Description
HG, U_i, SC_i, and S_j	HG, user, smart card, and sensor node, respectively
PID_i^{s1}, TID_i^s, and SID_j^s	Pseudonym for ith login, user, and sensor node, temporary identities, respectively
SK, K_u^s, and K_s^s	Session Key, HG, and sensor node keys, respectively
ID_i^s, PW_i^s Bio_i^s	Identity, password, and biometric of U_i
b_i^s, par_i^s	bio-metric key and reproduction parameters
T_x^s, and r_x^s	Timestamps and random numbers, where $x = 1, 2, 3, 4$
$\|$, $\oplus$, $H(.)$	Concatenation, XOR, and hash-function, respectively
$Gen(.)$, $Rep(.)$	FE bio-metric key generation, regeneration, reproduction function, respectively

A.2 Login and AKE phase

- U_i inputs its ID_i^s, PW_i^s, and imprints its bio-metric Bio_i^s at the terminal available at SC_i. U_i calculates $b_i^s = Rep(Bio_i^s)$, $u_i^s = D_i^s \oplus h(ID_i^s \| b_i^s)$, $TID_i^s = h(ID_i^s \| u_i^s)$, $HID_i^{ss} = h(TID_i^s \| K_u^s)$, and $B_i^{ss} = h(HPW_i^s \| HID_i^s)$. SC_i passes the local authentication if B_i^{ss} and the stored B_i^s are of the same value. Moreover, SC_i picks a random number r_i^s and calculates $PID_i^{s1} = C_i^{s1} \oplus h(TID_i^s \| HID_i^{ss})$, $R_i^s = h(TID_i^s \| PID_i^{s1} \| r_i^s)$, $M_i^s = r_i^s \oplus h(TID_i^s \| PID_i^{s1} \| T_1^s)$, and $M_{U,G}^s = h(TID_i^s \| HID_i^{ss} \| PID_i^s \| R_i^s \| T_i^s)$. Finally, SC_i dispatches a login message $\{T_1^s, PID_i^{s1}, M_i^s, M_{U_i,G}^s\}$ to HG via a public channel.

- After receiving the login message, HG validates the timestamp $| T_1^s - T_1^s | \leq \delta T^s$ and retrieves TID_i^s corresponding to PID_i^s. HG calculates $HID_i^{ss} = h(TID_i^s \| K_u^s)$, $r_i^s = M_i^s \oplus h(TID_i^s \| PID_i^{s1} \| T_1^s)$, $R_i^s = h(TID_i^s \| PID_i^{s1} \| r_i^s)$, and $M_{U,G}^{ss} = h(TID_i^s \| HID_i^{ss} \| PID_i^s \| R_i^s \| T_i^s)$. To validate the integrity of the received message, HG compares $M_{U,G}^s$ and $M_{U,G}^{ss}$. If both are of the same value, HG believes that U_i is a valid user. Otherwise, HG aborts this phase. HG selects a sensor for U_i, calculates $X_j^s = h(SID_j^s \| K_s^s)$, $M_G^s = R_i^{ss} \oplus h(X_j^s \| T_2^s)$, and $M_{G,S_j}^s = H(PID_i^{s1} \| SID_j^s \| X_{S_j} \| R_i^{ss} \| T_2^s)$ and sends a message $\{T_2^s, PID_i^{s1}, M_G^s, M_{G,S_j}^s, T_3^s\}$ to S_j through a public channel.

- S_j validates the received message's freshness by checking $| T_2^{'s} - T_2^s | \leq \delta T^s$, calculates $R_i^{ss} = M_G^s \oplus h(X_j^s \| T_2^s)$, and $M_{U,S_j}^{ss} = H(PID_i^{s1} \| SID_j^s \| X_{S_j} \| R_i^{ss} \| T_2^s)$. S_j compares M_{G,S_j}^{ss} and M_{G,S_j}^s to check the validity of message. Otherwise, S_j terminates the authentication phase. Moreover, S_j selects a random number r_j^s, calculates $M_j = r_{ss} \oplus h(X_{S_j} \| T_3)$, $R_j^s = h(SID_j^s \| r_j^s)$, $M_j^s = h(X_j^s \| T_3^s)$, $SK_{ij}^s = h(R_i^{ss} \| R_j^s)$ and $M_{S_j,HG}^s = h(PIDR_i^s \| SID_j^s \| X_{S_j} \| R_j^s \| SK_{ij} \| T_3)$, and dispatches a message $\{T_3^s, M_j^s, M_{S_j,G}^s\}$ to HG via an unprotected channel.

- After receiving the message from S_j, HG checks the condition $| T_3^{'s} - T_3^s | \leq \delta T^s$,

calculates $r_{ss} = M_j \oplus h(X_{S_j} \parallel T_3)$, $R_j^{ss} = h(SID_j^s \parallel r_j^s)$, $SK_{ij}^s = h(R_i^{ss} \parallel R_j^s)$, $M_{S_j,HG}^{ss} = h(PIDR_i^s \parallel SID_j^s \parallel X_{S_j} \parallel R_j^s \parallel SK_{ij} \parallel T_3)$, and compares both $M_{S_j,HG}^{ss}$ and $M_{S_j,HG}^s$. If both are equal, HG believes that this messages is from a legitimate node.

- HG picks a new pseudonym PID_i^{2s} and computes $C_i^{2s} = H(TID_i \parallel HID_i^{ss}) \oplus PID_i^{2s}$, p_i^{2s} $C_i^{s2} \oplus H(HID_i^{ss} \parallel T_4)$, $M_G^{ss} = R_j^{ss} \oplus h(PID_1^{s1} \parallel HID_i^{ss})$, and $M_{G,U_i}^s = h(PID_i^s \parallel HID_i^{ss} \parallel C_i^{s2} \parallel R_j^{ss} \parallel SK_{ij} \parallel T_4^s)$. Moreover, HG sends a message $\{T_4^s$, P_i^{s2}, M_G^{si}, $M_{G,U_i}^s\}$ to U_i.
- After receiving the message from HG, U_i ensures the message freshness by checking $\mid T_4^{'s} - T_4^s \mid \leq \delta T^s$. If the message is fresh then U_i computes $R_j^{ss} = M_G^{ss} \oplus h(PID_1^{s1} \parallel HID_i^{ss})$, $C_i^{s2} = p_i^{2s} \oplus H(HID_i^{ss} \parallel T_4)$, and $M_{G,U_i}^{ss} = h(PID_i^s \parallel HID_i^{ss} \parallel C_i^{s2} \parallel R_j^{ss} \parallel SK_{ij} \parallel T_4^s)$. Finally, U_i compares both M_{G,U_i}^{ss} and M_{G,U_i}^s to check the legitimacy of the received message. If the received message is valid then it updates C_i^{s1} with C_i^{s2}.

A.3 Security analysis of [1]

A.3.1 De-synchronization attack

De-synchronization attack is possible only when network entities need to have a matching state. The following steps demonstrate that the scheme is unprotected against de-synchronization attack.

- There are four messages exchanged during the AKE phase, include $\{T_1^s$, PID_i^{s1}, M_i^s, $M_{U_i,G}^s\}$, $\{T_2^s$, PID_i^{s1}, M_G^s, $M_{G,S_j}^s\}$, $\{T_3^s$, M_J^s, $M_{S_j,G}^s\}$, and $\{T_4^s$, P_i^{s2}, M_G^{si}, $M_{G,U_i}^s\}$. PID_i^{s1} is used to search TID_i^s in the database of HG. For every new AKE session in [1], HG updates PID_i^{s1} to PID_i^{s2}. HG sends PID_i^{s2} to U_i in message $\{T_4^s$, P_i^{s2}, M_G^{si}, $M_{G,U_i}^s\}$.
- Let an adversary $\mathcal{A}$ eavesdrop all communicated messages, which are exchanged during the AKE phase. Let $\mathcal{A}$ drops the message $\{T_4^s$, P_i^{s2}, M_G^{si}, $M_{G,U_i}^s\}$, which is sent from HG to U_i, prevents U_i from updating PID_i^{s1} to PID_i^{s2}. If the current AKE fails, U_i needs to use PID_i^{s1}. After receiving the new AKE request from U_i, HG searches PID_i^{s1} in its database. HG will not find any record related to PID_i^{s1} because in the last uncompleted AKE session, HG has updated PID_i^{s1} to PID_i^{s2}. In this way, the new AKE request received from U_i will fail. Therefore, $\mathcal{A}$ can effectuate the de-synchronization attack against the scheme of [1].

A.3.2 Design flaw

In the scheme of Shin *et al* [1], HG broadcasts the message $\{T_2^s$, PID_i^{s1}, M_G^s, $M_{G,S_j}^s\}$ to all sensor nodes deployed in the network. U_i does not specify the sensor node from which it is going to procure the information. Thus, all sensor nodes in the network will process the received message, which causes an extra computational overhead for every node. Therefore, U_i must intimate to HG for accessing information from a specific sensor node.

B Data Availability Statement

Minimal data set underlying the results described in this paper can be found at https://github.com/TanveerPhD/Minimal-data/blob/main/Data.ods

References

1. Shin S, Kwon T. A lightweight three-factor authentication and key agreement scheme in wireless sensor networks for smart homes. Sensors. 2019;19(9):2012.
